# High Seroprevalence of *Toxoplasma gondii* in Slovenian Wild Boars (*Sus scrofa*)

**DOI:** 10.3390/ani11113139

**Published:** 2021-11-03

**Authors:** Petra Bandelj, Diana Žele Vengušt, Rok Blagus, Aleksandra Vergles Rataj, Branko Krt

**Affiliations:** 1 Veterinary Faculty, University of Ljubljana, Gerbičeva ulica 60, SI-1115 Ljubljana, Slovenia; diana.zelevengust@vf.uni-lj.si (D.Ž.V.); aleksandra.verglesrataj@vf.uni-lj.si (A.V.R.); brane.krt@vf.uni-lj.si (B.K.); 2 Institute for Biostatistics and Medical Informatics, University of Ljubljana, Vrazov trg 2, SI-1104 Ljubljana, Slovenia; rok.blagus@mf.uni-lj.si; 3 Faculty of Sports, University of Ljubljana, Gortanova ulica 22, SI-1104 Ljubljana, Slovenia

**Keywords:** *Toxoplasma gondii*, wild boar, game meat, zoonosis, ELISA

## Abstract

**Simple Summary:**

*Toxoplasma gondii* is a parasite that can cause serious disease in humans, especially in pregnant women. This parasite is found in many animals and people can be infected by eating raw or undercooked meat. Wild boar are known to harbor this parasite; populations and habitats have increased in the past decade, as it also increased the consumption of venison. The European population of wild boar has a mean prevalence of 26%. In our study, we found that the prevalence in the Slovenian wild boar population is 62%, the highest in the world to date. The prevalence was influenced by age and weight, but not by gender. In conclusion, the hunting community should be made aware of the high risk of parasite exposure when dealing with wild boar meat.

**Abstract:**

*Toxoplasma gondii* is a zoonotic parasite of great public health concern. Wild boars could be considered an emerging source of toxoplasmosis in humans due to the popularity of venison and their increasing population. The aim of this study was to determine the seroprevalence of *T. gondii* in the Slovenian wild boar population and evaluate risk factors for human infection. Of 353 samples, 62% were positive for *T. gondii* using ELISA tests. This is the highest *T. gondii* seroprevalence reported to date in wild boar worldwide. The increase in prevalence with increasing age (*p* = 0.003) and weight (*p* = 0.002) were statistically significant, whereas gender was not (*p* = 0.781). Odds for being *T. gondii*-positive increased with age with the largest difference being between 2–3-year-old and 1–2-year-old animals (OR = 2.66, 95%CI: 1.03–6.85). Animals weighing 20–40 kg had a higher risk than animals weighing 0–20 kg (OR = 2.74, 95%CI: 1.21–6.20), whereas a further increase in the weight was not associated with increasing the odds. Due to the high Toxoplasma prevalence, the study concluded that the risk of exposure to *T. gondii* from handling raw or undercooked wild boar meat is high. Surveillance protocols should be established at the national level together with increased awareness within the hunting community.

## 1. Introduction

*Toxoplasma gondii* is the most widespread zoonotic protozoan parasite that has a wide range of warm-blooded hosts at its disposal. Sexual replication of *T. gondii* occurs in the intestine of felines, which are the end host of the protozoan parasite. Humans and most animals are considered intermediate hosts because they harbor tissue cysts containing tachyzoa [1]. It is estimated that one-third of the human population is infected with *T. gondii* [1]. Prenatal infections can lead to severe problems and even death [2], whereas postnatal toxoplasmosis, although rare, can cause eye and behavioral problems [3,4,5,6,7,8]. Humans can become infected in several ways: 1. by ingesting oocysts shed by cats, 2. by consuming food or water contaminated with oocysts, or 3. by consuming undercooked meat containing the encysted parasite [9,10,11,12]. Up to 50% of human toxoplasmosis cases are foodborne. Consumption of undercooked meat products containing *T. gondii* tissue cysts is the main risk factor for infection [13,14,15]. Although meat from domestic pigs is of greater concern [15], consumption of wild boar (*Sus scrofa*) meat has gained popularity [16,17]. This is mainly due to the successful spread of the species in the environment [17,18] and an increase in recreational hunting [16,17]. Wild boar, as omnivores, can contract toxoplasmosis by consuming food or water contaminated with sporulated oocysts or by ingesting infected tissues from other intermediate hosts [19]. The global seroprevalence of *T. gondii* in wild boar is estimated at 23%, with the highest seroprevalence being 26% and 32% in Europe and North America, respectively [17]. This high prevalence of toxoplasmosis makes wild boar a suitable biological model for the dynamic assessment of *T. gondii* in the environment where the wild boar population is constantly present [17,20]. Currently, there is no surveillance program for *T. gondii*-infected meat intended for human consumption [17], although the European Food Safety Authority (EFSA) has identified *T. gondii* as a relevant biological hazard that needs to be addressed [21]. The significant increase in the Central European wild boar population in recent years means an even greater risk of human and animal exposure to *T. gondii* [22].

The aim of this study was to determine the seroprevalence of *T. gondii* in the Slovenian wild boar population, to assess its role as a reservoir for human infection, and to evaluate which parameters recorded by hunters have a greater predictability value for a seropositive outcome.

## 2. Materials and Methods

### 2.1. Samples

Wild boar serum samples (n = 353) were collected in 2016 and 2017 from apparently healthy, free-ranging animals throughout Slovenia as part of the national surveillance program for brucellosis and African swine fever. Game wardens and hunters were asked to submit samples from animals shot during the regular annual harvest. Hunters were also instructed on procedures before sample collection and were provided with field sample kits. Shortly after death, blood samples were collected from the jugular vein or the heart. The samples were transported to the veterinary faculty at the University of Ljubljana, where the serum was obtained and stored at −20 °C until testing for Toxoplasma antibodies. Only animals whose harvest location, sex, age, and weight were recorded by the hunters were used for this study. The animals’ weights were determined by using a scale. The age of the animals was determined by tooth eruption, replacement, and wear.

The approval of the Ethics Committee/Welfare Authority was not required, as all samples were taken postmortem.

### 2.2. Methods

#### 2.2.1. Serological Methods

Two commercial test kits were used for the serological detection of *T. gondii* during the 2-year period. Samples from 2016 were tested with the ID Screen^®^ Toxoplasmosis Indirect Multi-Species kit (IDVET, Montpellier, France). Samples from 2017 were tested using pigtype^®^ Toxoplasma Ab (Qiagen, Hilden, Germany). Both ELISA kits are suitable for the detection of antibodies from wild boar serum and assays were performed according to the manufacturer’s instructions. Doubtful results were considered negative.

#### 2.2.2. Statistical Analyses

Data were summarized as frequencies (%). Differences between groups (positive vs. negative) for gender (male, female), age (4 categories: 0–1 year, 1–2 years, 2–3 years, >3 years), weight (5 categories: 0–20 kg, 20–40 kg, 40–60 kg, 60–80 kg, >80 kg) and region (12 regions) (Table 1) were tested using a chi-squared test with Yates continuity correction. The multivariate analysis was performed using a binary logistic regression. Random intercept by region was included in the model to account for the potential effect of the region (due to the large number of regions, region was not considered as a fixed effect). Due to high collinearity between age and weight, three multivariate models were fitted: (1) model including gender, age, and weight as fixed effects and region as a random effect, (2) model excluding age, and (3) model excluding weight. Results are presented as conditional odds ratios (ORs) with corresponding 95% confidence intervals (CIs). For all statistical analyses, effects were considered significant when the *p*-value was lower than 0.05. Statistical language R (version 3.6.1) was used for the analyses (R Core Team, 2019). R package lme4 was used to fit the models using 10 points per axis for evaluating the Gauss–Hermite approximation to the log-likelihood.

## 3. Results

Antibodies against *T. gondii* were detected in 220 of 353 (62%; CI 0.57–0.68) wild boar over a 2-year period. The univariate analysis showed seroprevalence in male and female wild boar of 63% and 61%, respectively, and the difference was not statistically significant (*p* = 0.781) (Table 1). However, seroprevalence was significantly associated with age (*p* = 0.003), weight (*p* = 0.002) and harvest location (*p* = 0.043) (Table 1). The results show an increase in prevalence from 51% in animals less than 1 year old to 83% in adult wild boar of 2–3 years old. Prevalence was 36% in animals weighing less than 20 kg, whereas it was over 70% in animals weighing more than 60 kg (Table 1). Weight and age had, adjusting for gender and region, similar predictive ability, AUC = 0.665 and 0.641 (AUC = area under the curve), respectively (Table 2). When adjusted for gender and region, 1–2-year-old animals had higher odds to be positive for *T.gondii* than 0–1-year-old animals (OR = 1.69. 95% CI: 1.02–2.77). Similarly, 2–3-year-old animals had higher odds than 1–2-year-old animals (OR = 2.66, 95% CI: 1.03–6.85), whereas the oldest group of animals (more than 3 years old) had a smaller, but not statistically significant, odds than animals aged 2–3 years (OR = 0.50, 95% CI: 0.15–1.63).

**Figure 1:**
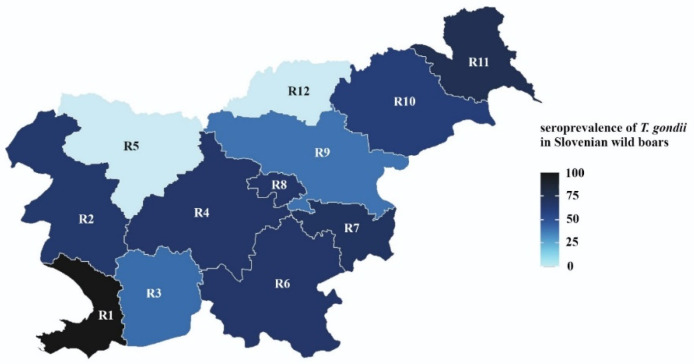
Seroprevalence of *T. gondii* in Slovenian wild boar (%). Regions (R): R1—obalno kraska, R2—goriska, R3—primorsko notranjska, R4—osrednjeslovenska, R5—gorenjska, R6—jugovzhodna Slovenija, R7—posavska, R8—zasavska, R9—savinjska, R10—podravska, R11—pomurska, R12—koroska.

After adjusting for gender and region, animals weighing 20–40 kg had higher odds than animals weighing 0–20 kg (OR = 2.74, 95% CI: 1.21–6.20), whereas higher weight was no longer significantly associated with further increasing the odds for being *T.gondii*-positive (OR = 1.42, 95% CI: 0.81–2.48, OR = 1.58, 95% CI: 0.73–3.37 and OR = 0.80, 95% CI: 0.28–2.25 for 40–60 kg vs. 20–40 kg, 60–80 kg vs. 40–60 kg and over 80 kg vs. 60–80 kg, respectively). Due to high collinearity between age and weight (*p* < 0.001), both effects were, adjusting for gender and region, not significant when age and weight were simultaneously included in the model (*p* = 0.301 and 0.095 for age and weight, respectively) (Figure 1).

## 4. Discussion

Wild boar meat is a potential source of *T. gondii* infection in humans. Due to the recent significant increase in the wild boar population in Europe and the expansion of its habitat, it poses an increasing public health concern [13,15,17,23,24,25]. This study was carried out to assess the status of *T. gondii* prevalence in the Slovenian wild boar population, to determine its potential as a reservoir for human infection, and to evaluate potential risk factors based on sex, age, weight, and harvest location. This is the first report on the seroprevalence of *T. gondii* in wild boar in Slovenia and the first to consider weight to assess the probability of infection.

Samples were collected from all over Slovenia over a two-year period and tested for the presence of *T. gondii* antibodies. The overall seroprevalence was determined at 62%, which is the highest worldwide seroprevalence recorded to date. Although the seroprevalence of *T. gondii* in the European wild boar population is estimated at 26%, as noted by Rostami et al. [17], there are countries with a similarly high prevalence as in our study. Romania and Sweden have a *T. gondii* seroprevalence in wild boar of 57% and 50%, respectively [19,26]. In Spain, France, the Czech Republic, and the Slovak Republic, the prevalence is around 40% [22,27,28,29,30]. The high prevalence in Slovenian wild boar is surprising. Although Slovenia (46°8′57″ N/14°59′34″ E) has an ideal Mediterranean/Continental/pre-Alpine climate that allows successful survival of *T. gondii* and wild boar [1,17,31], only 55% of the territory is populated with wild boar [31]. Steep and rough terrain in mountainous regions is not an optimal habitat for wild boar [31], which explains why only a small number of animals were sampled in some regions. The results showed statistically significant differences in the prevalence of *T. gondii* due to regions, but because of too many regions combined with a low number of sampled animals in some regions, we could not estimate its effect by the model. 

There was no difference between *T. gondii* seroprevalence in male (63%) and female (61%) wild boar. This is consistent with other studies showing that gender is not associated with the likelihood of infection with *T. gondii* [25,32]. In agreement with previously published studies, increasing age is also associated with a higher prevalence of Toxoplasma antibodies [17,33,34]. The general increase in the prevalence of *T. gondii* with age was expected: in our study, the rate ranged from 51% in the youngest group to 83% in the older group of wild boars, as animals have more opportunities to encounter the parasite over time [17,33,34]. Our samples from wild boar were divided into four age groups as previously reported by Gauss et al. [27], but we used the age categorization of Bier et al. [25] in the younger group (0–1 year instead of 0–0.5 years and 0.5–1 year). Using four age groups instead of only two or three [25,35] should provide a more accurate insight into the influence of age on the prevalence of *T. gondii* in adult wild boar (1–2, 2–3, and over 3 years old) and remain practical from the hunter’s perspective. In our study, there was a significant increase in the group of 1–2-year-old and 2–3-year-old wild boar compared with the group of 0–1-year-olds and 1–2-year-olds, respectively. Interestingly, the prevalence of *T. gondii* reached a plateau after reaching three years of age. Regardless of the number of age groups used (2–4), some studies failed to show statistical significance of age-related seroprevalence in wild boar, although prevalence increased with age [25,27,35,36]. The prevalence of *T. gondii* in wild boar also increased with increasing weight, from 36% in animals weighing less than 20 kg to 76% in animals weighing 60–80 kg. A significant increase in prevalence was observed when animals weighing 20–40 kg were compared with animals weighing less than 20 kg, whereas further increases in weight were not associated with further increases in odds. To date, no data have been reported on the prevalence of *T. gondii* in relation to the weight of wild boar, which is understandable given the strong association between increasing weight and age. However, weight is accurately measured by experienced and less experienced hunters, whereas age can be less accurately determined if the hunter is not experienced. Since both parameters are usually noted by hunters, we decided to evaluate which of the two variables is a better predictor of a positive *T. gondii* outcome. Age and weight were evaluated together in Model 1 and separately in Models 2 (weight) and Model 3 (age). All models had similar predictive power, indicating that both variables (age and/or weight) can be used equally to estimate the risk of exposure to the parasite. To the best of the authors’ knowledge, this is the first study to report weight-dependent *T. gondii* seroprevalence in wild boar.

In Slovenia, the prevalence of toxoplasmosis in women of childbearing age has decreased in recent decades, from over 50% in the 1980s to about 25% in the new millennium [12,37]. This is probably due to improved hygiene, lower *T. gondii* infection in animals from intensive farming, and increased consumption of frozen meat [12]. However, the Slovenian wild boar population has increased significantly over the last decade and is expected to increase further as land is still available for habitat expansion [31]. The high population density of wild boar and the highest *T. gondii* seroprevalence worldwide to date means that the Slovenian wild boar population could be an important source of *T. gondii* infection in humans—particularly for those who handle and consume raw or undercooked venison, which is becoming increasingly popular [25]. The high seroprevalence does not mean that all seropositive animals are infected with tissue cysts [25,28,38]. A study by Richomme et al. [28] described that only half of the seropositive animals had infectious tissue cysts. If we take this information into account, the probability of becoming infected with *T. gondii* through handling and consuming raw wild boar meat could be one in three for people living in the territory of Slovenia. Because of the public health importance, surveillance protocols for *T. gondii* in wild boar and other game animals should be considered at the national level. A follow-up study with other game animals and potential *T. gondii* sources would be helpful to better determine the reasons for the high seroprevalence in the wild boar population and its dynamics in the environment.

## 5. Conclusions

The Slovenian wild boar population has the highest prevalence of *T. gondii* antibodies in the world to date. Weighing and/or accurate determination of the age of the animal may have good predictive value for *T. gondii* infection. The hunting community is at high risk of *T. gondii* infection because its members regularly handle uncooked raw wild boar meat. Therefore, additional efforts should be made to educate people in the hunting community about the impact of the parasite and its implications for its more susceptible members.

## Figures and Tables

**Table 1:** Seroprevalence of *T. gondii* in wild boar based on sex, age, weight, and region.

Wild Boar	Tested Animals (%)	*T. gondii* Positive (%)	*p* *
Gender			0.781
male	209 (59)	132 (63)	
female	144 (41)	88 (61)	
Age (years)			0.003
0–1	125 (35)	64 (51)	
1–2	162 (46)	105 (65)	
2–3	35 (10)	29 (83)	
>3	31 (9)	22 (71)	
Weight (kg)			0.002
0–20	33 (9)	12 (36)	
20–40	137 (39)	79 (58)	
40–60	99 (28)	66 (67)	
60–80	55 (16)	42 (76)	
>80	29 (8)	21 (72)	
Region			0.043
1 obalno kraska	2 (1)	2 (100)	
2 goriska	20 (6)	13 (65)	
3 primorsko notranjska	43 (12)	18 (42)	
4 osrednjeslovenska	18 (5)	12 (67)	
5 gorenjska	0	0	
6 jugovzhodna slovenija	129 (36)	87 (67)	
7 posavska	30 (8)	21 (70)	
8 zasavska	12 (3)	8 (67)	
9 savinjska	20 (6)	8 (40)	
10 podravska	55 (16)	33 (60)	
11 pomurska	24 (7)	18 (75)	
12 koroska	0	0	

Data are frequencies (%), * *p*-value from a chi-squared test with continuity correction.

**Table 2:** Risk factors for the prevalence of *T. gondii* in wild boar. The results are OR (95% confidence intervals) and *p*-values.

Wild Boar	Model 1 (AUC = 0.664)	Model 2 (AUC = 0.665)	Model 3 (AUC = 0.641)
Gender	*p* = 0.963	*p* = 0.911	*p* = 0.915
male vs. female	1.01 (0.63–1.61)	1.03 (0.65–1.63)	0.98 (0.61–1.54)
Age (years)	*p* = 0.301		*p* = 0.004
1–2 vs. 0–1	1.24 (0.70–2.19) [*p* = 0.454]	/	1.69 (1.02–2.77) [*p* = 0.038]
2–3 vs. 1–2	2.19 (0.78–6.09) [*p* = 0.132]	/	2.66 (1.03–6.85) [*p* = 0.042]
>3 vs. 2–3	0.47 (0.13–1.65) [*p* = 0.238]	/	0.5 (0.15–1.63) [*p* = 0.249]
Weight (kg)	*p* = 0.094	*p* = 0.001	
20–40 vs. 0–20	2.54 (1.11–5.84) [*p* = 0.028]	2.74 (1.21–6.2) [*p* = 0.015]	/
40–60 vs. 20–40	1.22 (0.66–2.23) [*p* = 0.52]	1.42 (0.81–2.48) [*p* = 0.216]	/
60–80 vs. 40–60	1.41 (0.61–3.24) [*p* = 0.417]	1.58 (0.73–3.38) [*p* = 0.238]	/
>80 vs. 60–80	0.76 (0.24–2.36) [*p* = 0.64]	0.8 (0.28–2.25) [*p* = 0.666]	/

## Data Availability

The data presented in this study are available on request from the corresponding author.
